# A Ternary Dumbbell Structure with Spatially Separated Catalytic Sites for Photocatalytic Overall Water Splitting

**DOI:** 10.1002/advs.201903568

**Published:** 2020-07-14

**Authors:** Bocheng Qiu, Lejuan Cai, Ning Zhang, Xiaoming Tao, Yang Chai

**Affiliations:** ^1^ Department of Applied Physics The Hong Kong Polytechnic University Hung Hom Kowloon Hong Kong 999077 P. R. China; ^2^ The Hong Kong Polytechnic University Shenzhen Research Institute Shenzhen 518057 P. R. China; ^3^ Institute of Textiles and Clothing The Hong Kong Polytechnic University Hung Hom Kowloon Hong Kong 999077 P. R. China

**Keywords:** charge transfer, dual cocatalysts, nanodumbbells, overall water splitting, spatially separated sites

## Abstract

Solar‐driven overall water splitting based on metal sulfide semiconductor photocatalysts remains as a challenge owing to the strong charge recombination and deficient catalytic active sites. Additionally, significant inhibition of back reactions, especially the oxidation of sulfide ions during the photocatalytic water oxidation catalysis, is an arduous task that requires an efficient photogenerated hole transfer dynamics. Here, a ternary dumbbell‐shaped catalyst based on RuO_2_/CdS/MoS_2_ with spatially separated catalytic sites is developed to achieve simultaneous production of hydrogen and oxygen under simulated solar‐light without any sacrificial agents. Particularly, MoS_2_ nanosheets anchored on the two ends of CdS nanowires are identified as a reduction cocatalyst to accelerate hydrogen evolution, while RuO_2_ nanoparticles as an oxidation cocatalyst are deposited onto the sidewalls of CdS nanowires to facilitate oxygen evolution kinetics. The density functional theory simulations and ultrafast spectroscopic results reveal that photogenerated electrons and holes directionally migrate to MoS_2_ and RuO_2_ catalytic sites, respectively, thus achieving efficient charge carrier separation. The design of ternary dumbbell structure guarantees metal sulfides against photocorrosion and thus extends their range in solar water splitting.

## Graphical abstract

The ternary dumbbell‐like RuO_2_/CdS/MoS_2_ photocatalyst with selective attachment of reduction and oxidation cocatalysts enables water splitting into H_2_ and O_2_. The critical aspect is the spatially separated distribution of MoS_2_ and RuO_2_ dual cocatalysts, which not only achieves photogenerated electron–hole pairs separation, but also protects CdS from photocorrosion by holes.

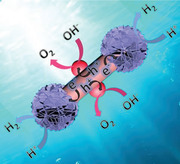

The global energy demand continues to rise, which presents inextricably serious challenges to environment issues, and drives the quest for clean and renewable energy sources alternative to fossil fuels.^[^ ^1^, ^2^, ^3^ ^]^ It has been regarded as a simple and cost‐effective method to produce clean hydrogen fuel by photocatalytic overall water splitting,^[^ ^4^, ^5^, ^6^ ^]^ comparable to fossil‐fuel‐derived hydrogen due to its simplicity and low cost.^[^ ^7^, ^8^, ^9^ ^]^ However, the photocatalysts for water splitting in previously reported literature still face grand challenges: 1) most of photocatalysts suffer from weak visible light response owing to their large bandgap;^[^ ^10^, ^11^, ^12^ ^]^ 2) O_2_ evolution from semiconductor photocatalysts is difficult due to multiple electrons and protons transfer process for the formation of O—O bond (1.23 eV).^[^ ^13^, ^14^, ^15^, ^16^ ^]^ In this regard, it is vital to develop narrow‐bandgap and low‐cost photocatalysts capable of decomposing water into hydrogen and oxygen to maximize the photoconversion efficiency.

Transitional metal chalcogenides is a promising candidate for efficient solar energy harvesting due to their simple chemical composition, narrow bandgap, and appropriate band structure.^[^ ^17^, ^18^, ^19^, ^20^ ^]^ The structure of 1D metal chalcogenides with reduction‐cocatalyst tip possesses a few unique advantages in photocatalytic water splitting, such as fully exposed active sites, effective hole localization, and long‐distance electron transport.^[^ ^21^, ^22^, ^23^, ^24^, ^25^, ^26^ ^]^ For instance, 1D CdS nanorods with Pt nanoparticles (NPs) at the end of nanorods can show impressive photocatalytic H_2_ evolution performance with a high quantum efficiency of 9.6% under the light illumination with the wavelength of 455 nm.^[^ ^27^ ^]^ This optimized reduction‐cocatalyst‐tipped semiconductor nanostructure fully exploits the benefits of interfacial charge separation between photogenerated electrons transferring to Pt cocatalyst and holes remaining on the CdS nanorods, thus resulting in longer electron lifetime. The improvement of the photogenerated electron lifetime is closely associated with the introduction of sacrificial electron donors. With the absence of sacrificial electron donors, the photoinduced holes accumulated on the sidewalls of 1D metal sulfide tend to oxidize lattice sulfide ions rather than involve in oxygen evolution.^[^ ^28^, ^29^ ^]^ Consequently, an efficient hole transfer is a key step to achieve high‐efficiency solar‐to‐fuel conversion.

Recently, researchers employed metal‐based molecular cocatalysts as holes collector and investigated the effect of tunable ligand structure on water oxidation activity.^[^ ^30^, ^31^ ^]^ Nevertheless, the metal‐based molecular cocatalysts require complicated organic synthetic process and exhibit poor stability as a result of hole‐induced ligand oxidation.^[^ ^32^, ^33^ ^]^ Compared with metal‐based molecular cocatalysts, non‐photoresponsive metal oxides, such as RuO_2_ and IrO_2_, are alternative oxidation cocatalysts with low resistivity and high corrosion resistance.^[^ ^34^, ^35^, ^36^ ^]^ Therefore, we can expect that the deposition of metal oxides cocatalysts onto the sidewalls of 1D metal sulfide structure can effectively transfer multiple holes from sulfide to oxidation cocatalysts sites, which is indispensable for water oxidation.

In this work, we design and fabricate a nanodumbbell structure photocatalyst based on CdS nanowires anchored with MoS_2_ (electron collector) and RuO_2_ (hole collector) dual cocatalysts to successfully achieve simultaneous H_2_ and O_2_ evolution (**Figure** **1**a). MoS_2_ NSs as electron trappers are decorated at the both tips of CdS nanowires, while RuO_2_ NPs as hole collectors are deposited on the sidewalls of CdS nanowires. The photogenerated electrons and holes on the CdS nanowires flow to tips and sidewalls, respectively, and charges accumulated on the corresponding cocatalysts subsequently take part in overall water splitting (Figure 1b).

**Figure 1:**
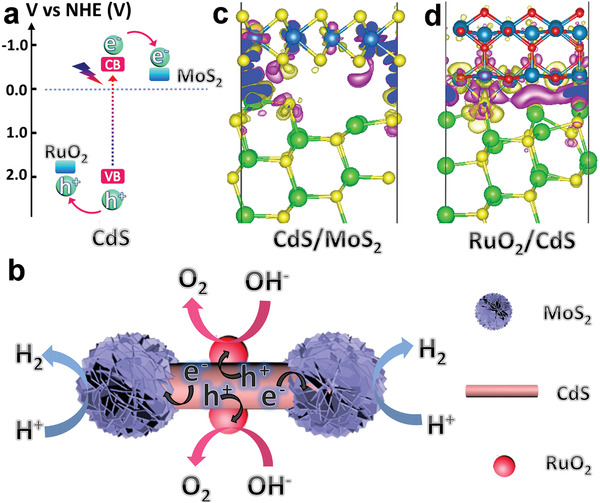
a) The feasibility analysis of solar‐driven overall water splitting on CdS photocatalysts decorated with reduction (MoS_2_) and oxidation (RuO_2_) cocatalysts. b) The migration path of photogenerated electrons and holes in photocatalytic water‐splitting reaction driven by RuO_2_/CdS/MoS_2_ nanodumbbells. Differential charge density [Δ*ρ* = *ρ*(A/CdS) − *ρ*(A) − *ρ*(CdS) (A = MoS_2_ or RuO_2_)] of c) CdS/MoS_2_ and d) RuO_2_/CdS, respectively. Electron excess and deficiency were displayed as purple and light yellow isosurfaces, respectively, with the level of 0.003 e Å^−3^.

The band structure of CdS nanowires was first investigated to analyze the possibility of photocatalytic overall water splitting (Figure S1, Supporting Information). The bandgap of CdS is determined to be 2.12 eV from the Tauc plot (Figure S1a,b, Supporting Information), and its valence band (VB) potential is calculated to be 1.88 eV from X‐ray photoelectron spectroscopy (XPS) (Figure S1c, Supporting Information). The conduction band (CB) position is thus estimated at −0.24 eV. Electronic band structure of CdS (Figure S1d, Supporting Information) further shows that the reduction level for H_2_ is positioned below the CB of CdS, while the oxidation level for H_2_O to H_2_O_2_ or O_2_ is above the VB of CdS, which suggests thermodynamical possibility for photocatalytic overall water splitting.^[^ ^37^ ^]^ After introducing dual cocatalysts onto CdS nanowires, we hypothesize that electron coupling effects are existed at both CdS/MoS_2_ and CdS/RuO_2_ interfaces, which is essential for photogenerated electrons and holes transfer. We performed density function theory (DFT) simulations to investigate the coupling effect and charge transfer route at the interfaces. These two heterostructures based on CdS/MoS_2_ and RuO_2_/CdS are formed by the strong chemical bonding interactions, due to the high binding energies of —0.30 and −1.08 J m^−2^, respectively. Furthermore, Figure 1c,d shows high differential charge density at the CdS/MoS_2_ and RuO_2_/CdS junction region, indicating the remarkable charge transfer occurs at the interfaces. Clearly, the outmost Cd atoms of CdS donate electrons to the S atoms of MoS_2_ at the MoS_2_/CdS heterostructure interface (Figure 1c). Bader charge analysis further indicates 0.82 electrons transfer from CdS to MoS_2_. For the RuO_2_/CdS system (Figure 1d), a significant reconstruction of interfacial atoms from CdS is caused by the polarization effect of RuO_2_, resulting in more complicated electrons redistribution at the interface of RuO_2_/CdS. The charge transfer (0.62 electrons) calculated from Bader charge reveals the formation of positively charged RuO_2_ and negatively charged CdS at the RuO_2_/CdS interface, indicating the surface of RuO_2_ attracts holes from the adjacent CdS. Our calculations suggest that the strong interface coupling on both CdS/MoS_2_ and RuO_2_/CdS enables efficient interfacial charge carrier transfer in ternary RuO_2_/CdS/MoS_2_ structure, which is essential for high photocatalytic performance.

We designed a synthetic strategy to fabricate the ternary RuO_2_/CdS/MoS_2_ photocatalyst with nanodumbbells structure (**Figure** **2**a). Amine‐functionalized CdS nanowires with average diameter of 60–70 nm were first prepared through a solvothermal approach by using ethanediamine as the chelating agent, as confirmed by scanning electron microscope (SEM) (Figure S2a,b, Supporting Information) and transmission electron microscope (TEM) images (Figure S2c,d, Supporting Information). X‐ray diffraction (XRD) pattern shows characteristic peaks of CdS with hexagonal Wurtzite crystal structure (Figure S3, Supporting Information). The MoS_2_ NSs were further decorated with the two ends of amine‐functionalized CdS nanowires (CdS/MoS_2_) by hydrothermal treatment with Na_2_MoO_4_ and thioacetamide as the MoS_2_ precursors in the mixture of H_2_O and diethylenetriamine. The SEM and TEM images (Figure S4, Supporting Information) show that MoS_2_ NSs are selectively and symmetrically grown on the two tips of CdS nanowire to form a well‐defined nanodumbbell structure. After MoS_2_ NSs affixed CdS nanowire tips, the surface‐modified —NH_2_ groups still remain at the sidewalls of CdS nanowires, which is confirmed by Fourier transform infrared spectra (Figure S5, Supporting Information). The strong coordination between Ru^3+^ and —NH_2_ groups ensures that the dispersion of Ru^3+^ ions on the sides of CdS/MoS_2_.^[^ ^38^ ^]^ Following a thermal treatment process, RuO_2_ NPs were selectively formed on the sidewalls of CdS nanowires. The morphology characterizations manifest that the prepared RuO_2_/CdS/MoS_2_ sample still maintains symmetrical tip‐coated structure (Figure 2b,c), and some ultrasmall RuO_2_ NPs are precisely located on the sidewalls of CdS nanowires (Figure 2d,e). More closer TEM observations (Figure 2f,g,h) on the three selected regions (Figure 2e) and their corresponding energy dispersive X‐ray (EDX) spectra (Figure S6, Supporting Information) show that the RuO_2_ NPs with average size of 4 nm are selectively loaded on the sidewalls of CdS rather than the MoS_2_ tips. A high resolution TEM (HRTEM) image of the sidewall of RuO_2_/CdS/MoS_2_ (Figure 2i) reveals that two sets of lattice distances of 0.179, and 0.320 nm, assigning to (200) plane of CdS and (110) planes of RuO_2_, respectively. As for the end of RuO_2_/CdS/MoS_2_ (Figure 2j), the interplanar spacings of 0.179 and 0.625 nm are ascribed to (200) plane of CdS and (002) plane of MoS_2_, respectively. The spatially separated distribution of MoS_2_ and RuO_2_ on CdS is further confirmed by high‐angle annular dark‐field scanning transmission electron microscopy and elemental mapping. As displayed in Figure 2k,m, MoS_2_ NSs and RuO_2_ NPs are preferentially formed at the tips and sidewalls of CdS nanowires, respectively. For comparison, RuO_2_/CdS nanowires were also synthesized by depositing RuO_2_ onto the sidewalls of CdS nanowires, as confirmed by SEM, TEM, and XRD pattern (Figure S7, Supporting Information).

**Figure 2:**
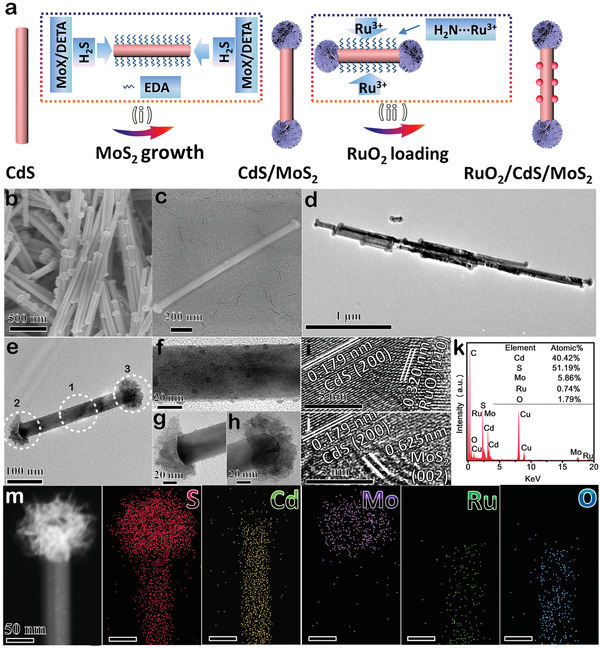
a) Schematic illustration of RuO_2_/CdS/MoS_2_ nanodumbbell preparation process (i: selective growth of MoS_2_ NSs at the two ends of CdS nanowires; ii: anchoring RuO_2_ NPs on the sidewalls of CdS/MoS_2_ nanodumbbells). b,c) SEM images and d,e) TEM images of RuO_2_/CdS/MoS_2_ nanodumbbells. f–h) The corresponding magnified TEM images for selected three part (1,2,3) in (e). i,j) HRTEM images of RuO_2_/CdS/MoS_2_ nanodumbbells. k) EDX spectrum and m) the corresponding element mapping of S, Cd, Mo, Ru, O.

The crystal structure and element composition of RuO_2_/CdS/MoS_2_ are confirmed by XRD patterns and XPS spectra. The XRD pattern (**Figure** **3**a) indicates that as‐prepared sample still preserves hexagonal Wurtzite‐structured phase CdS (JCPDS No. 65‐3414), and no characteristic peaks of MoS_2_ or RuO_2_ are observed due to their relative low Mo (9.31 wt%) and Ru (0.96 wt%) concentration, which is confirmed by the inductively coupled plasma mass spectrometry (ICP‐MS) (Table S1, Supporting Information). XPS survey spectrum (Figure S8, Supporting Information) shows the coexistence of Cd, S, Mo, Ru, and O elements, indicating successful deposition of MoS_2_ and RuO_2_ on CdS. The high resolution XPS signal (Figure 3b) of S 2p can be well deconvoluted into two separate peaks located at 162.8 and 161.6 eV, corresponding to S 2p_1/2_ and S 2p_3/2_ in the form of S^2−^, respectively.^[^ ^39^, ^40^ ^]^ The high resolution Ru 3p XPS spectrum (Figure 3c) exhibits the doublets for Ru 3p_1/2_ and Ru 3p_3/2_ peaks at 484.5 and 461.7 eV, respectively, as expected for RuO_2_.^[^ ^41^ ^]^ With regard to the XPS spectrum of O 1s (Figure 3d), two discrete peaks at 530.9 and 531.9 eV are indexed to Ru—O—Ru and Ru—O—H, and the other one at 532.8 eV is attributed to C—O bond, which originates from the surface oxygenated groups.^[^ ^42^, ^43^ ^]^

**Figure 3:**
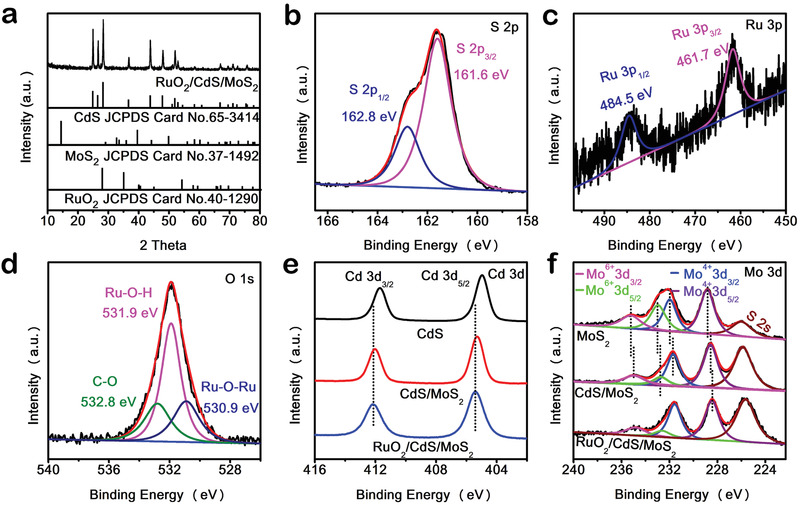
a) XRD pattern of RuO_2_/CdS/MoS_2_ nanodumbbells. High resolution XPS spectra of S 2p b), Ru 3p c), and O 2p d) of RuO_2_/CdS/MoS_2_ nanodumbbells. e) High‐resolution Cd 3d XPS spectra of CdS, CdS/MoS_2_, and RuO_2_/CdS/MoS_2_. f) High‐resolution Mo 3d XPS spectra of MoS_2_, CdS/MoS_2_, and RuO_2_/CdS/MoS_2_.

To achieve fast interfacial charge transfer at the heterostructure interface, the formation of intimate electron coupling interface is of great importance. Compared to those of CdS, Cd 3d peaks for CdS/RuO_2_ demonstrate negative shift of 0.1 eV (Figure S9a, Supporting Information), suggesting more free electrons accumulation at CdS caused by the holes transfer from CdS to RuO_2_ after their combination, which is in agreement with the DFT simulation results. Similarly, the S 2p peaks for CdS/RuO_2_ also display negative shift in binding energy (Figure S9b, Supporting Information). However, an obvious shift of 0.3 eV to high binding energy region can be also observed in Cd 3d XPS spectrum of CdS/MoS_2_ as compared to that of CdS (Figure 3e), unraveling electron transfer from CdS to MoS_2_.^[^ ^44^ ^]^ Moreover, a slight shift of 0.1 eV toward the high binding energy is observed after further RuO_2_ loading, suggesting a strong electron coupling effect between CdS and RuO_2_, which contributes to the charge transfer from CdS to RuO_2_. Meanwhile, Mo 3d peaks for MoS_2_ show continuous shift toward low binding energy after hybridization with MoS_2_ and RuO_2_ in turn (Figure 3f).

Ultraviolet visible (UV–vis) diffuse reflectance spectra were employed to investigate the light harvesting capability of the as‐prepared samples. As displayed in **Figure** **4**a, the light absorption of CdS/MoS_2_ nanodumbbells is clearly increased in the visible light region as compared to CdS nanowires, which is attributed to the black color of MoS_2_.^[^ ^45^ ^]^ After further RuO_2_ NPs depositing, a stronger light response can be observed for RuO_2_/CdS/MoS_2_. It is also noted that negligible shift in absorption edge of RuO_2_/CdS/MoS_2_ can be observed, which manifests that Ru, O, or Mo atom is undoped into the crystal lattice of CdS. The charge separation and transfer behavior of the as‐made samples were studied by transient photocurrent response spectroscopy, electrochemical impedance spectra (EIS), and photoluminescence (PL) spectroscopy. As displayed in Figure 4b, RuO_2_/CdS/MoS_2_ shows the highest photocurrent intensity, suggesting enhancement of charge separation efficiency by introducing spatially separated MoS_2_ and RuO_2_ dual cocatalysts. Furthermore, the EIS spectra (Figure S10, Supporting Information) demonstrate that the semicircle diameter of RuO_2_/CdS/MoS_2_ is the smallest one among all the samples, indicating the optimal interfacial charge‐carrier transfer ability on the surface of RuO_2_/CdS/MoS_2_. In addition, PL quenching experiments were conducted to investigate the electron transfer dynamics. The most significant quenching of PL is observed by the decoration of dual cocatalysts, testifying the prolonged lifetime of photogenerated charge carrier in RuO_2_/CdS/MoS_2_. To better understand the spatial separation behavior of photoinduced charge carriers in RuO_2_/CdS/MoS_2_, two photodeposition reactions, reduction of Au^3+^ with lactic acid as hole collectors and oxidation of Mn^2+^ with IO_3_^−^ as electron acceptors were carried out, respectively. As indicated in Figure S11 (Supporting Information), Au NPs were selectively loaded on the MoS_2_ tips, suggesting that the photogenerated electrons tend to migrate to MoS_2_ sites for reduction reaction. By contrast, MnO*_x_* NSs derived from Mn^2+^ oxidation were deposited only on RuO_2_ part (Figure S12, Supporting Information), revealing the photogenerated holes are inclined to accumulate on RuO_2_ sites for oxidation reaction.

**Figure 4:**
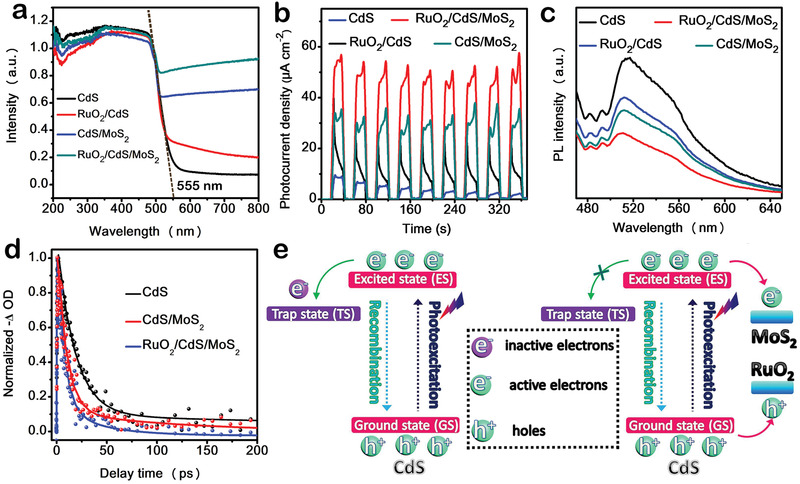
a) UV–vis diffuse reflectance spectra, b) Transient photocurrents, and c) PL spectra of CdS, RuO_2_/CdS, CdS/MoS_2_, and RuO_2_/CdS/MoS_2_. d) Normalized fs‐TA decay kinetics (dotted line) with a biexponential function fitting curves (solid lines). e) Schematic representation of proposed charge transfer and recombination routes in CdS and RuO_2_/CdS/MoS_2_.

To further ascertain the nature of the photoinduced charge carrier transfer interaction, femtosecond transient absorption (Fs‐TA) spectroscopy was carried out by employing pump–probe technique using 400 nm laser as a pump pulse and a white light continuum of 440–700 nm as the probe pulse. Upon 400 nm excitation, these three samples of CdS, CdS/MoS_2_, and RuO_2_/CdS/MoS_2_ show the distinctly negative absorption peak at 475 nm (Figure S13, Supporting Information), which is attributed to photobleaching of the ground state (GS) absorption.^[^ ^46^ ^]^ Transient absorption (TA) kinetic decay traces of the three samples are fitted with biexponential decay, and the fitting parameters are summarized in Table S2 (Supporting Information). Upon light irradiation, photoinduced electrons can be first excited from GS to excited state (ES) followed by two fast processes involving charge recombination from ES to GS and charges trapping from ES to inactive trap states (TS). It is noted that the electrons on TS are inactive and not involved into photocatalytic reaction.^[^ ^47^ ^]^ For CdS nanowires, the decay observed within 10 ps (*τ*_1_, Table S2, Supporting Information) is assigned to the active charge recombination from ES to GS, while the lifetime of 350.8 ps (*τ*_2_, Table S2, Supporting Information) is attributed to the charge transfer process from ES to TS. Regarding CdS/MoS_2_, the fast decay component of 8.1 ps (*τ*_1_, Table S2, Supporting Information) is mainly ascribed to the charge recombination from ES to GS in CdS. The lifetime of 123.5 ps (*τ*_2_, Table S2, Supporting Information) is attributed to the charge transfer from ES of CdS to MoS_2_ rather than from ES to TS in CdS, because the efficient electron capture ability of MoS_2_ prevents photogenerated electrons from trapping into inactive TS in CdS. After the decoration of RuO_2_ on the sidewalls of CdS/MoS_2_ nanodumbbells, the lifetime (*τ*_2_, Table S2, Supporting Information) is further reduced to 40.8 ps, revealing that the introduction of RuO_2_ promotes electron transfer process from ES of CdS to MoS_2_, which accords with the PL results.

The photocatalytic activity of the as‐prepared samples were evaluated by hydrogen and oxygen evolution from water‐splitting reaction under simulated solar light irradiation (a 300 W Xe lamp equipped with AM1.5 optical filter) without any sacrificial reagents. As indicated in **Figure** **5**a, both bare CdS and RuO_2_/CdS nanowires show negligible H_2_ production activity, while CdS/MoS_2_ nanodumbbells display moderate H_2_ evolution activity (0.7 µmol h^−1^) due to the decreased charge recombination rate in terms of the electron transfer from CdS to MoS_2_. After further depositing of RuO_2_ NPs, the hydrogen evolution rate of RuO_2_/CdS/MoS_2_ nanodumbbells can be remarkably improved and reach up to 5.2 µmol h^−1^, and O_2_ evolution proceeds continuously at a rate of 1.1 µmol h^−1^. It should be also noted that RuO_2_/CdS/MoS_2_ achieves photocatalytic water splitting into H_2_ (2.7 µmol h^−1^) and O_2_ (0.6 µmol h^−1^) under visible light irradiation (*λ*>420 nm), indicating the efficient solar energy harvesting of RuO_2_/CdS/MoS_2_. Besides, control experiments show that no O_2_ evolution was detected by gas chromatography (GC) when CdS/MoS_2_ was used as photocatalyst over a 5 h reaction period, which indicates a well‐established photocorrosion reaction between S^2−^ and the holes remaining on the sides for CdS/MoS_2_ nanodumbbells. The TEM observations (Figure S14a, Supporting Information) and XRD patterns (Figure S14b, Supporting Information) of CdS/MoS_2_ after the water‐splitting reaction further prove that photoinduced holes can readily oxidize the lattice S^2−^ ions of CdS.^[^ ^48^ ^]^ Similar phenomenon is also observed for bare CdS nanowires (Figure S14c,d, Supporting Information). Furthermore, the linear sweep voltammetry (LSV) curve of the RuO_2_/CdS/MoS_2_ nanodumbbells demonstrates an obvious reduction of the S^2−^ ions oxidation peak along with the decreased overpotential of oxygen evolution as compared to that of the CdS/MoS_2_ nanodumbbells (Figure 5b), indicating the holes transfer from CdS to RuO_2_ in RuO_2_/CdS/MoS_2_ and the minimization of S^2−^ ions oxidation during water oxidation. Besides, the dependence of photocatalytic performance on the loading amounts of MoS_2_ and RuO_2_ was illustrated in Figure S15 (Supporting Information). It can be observed that the nominal loading amounts of MoS_2_ and RuO_2_ were 15.52 and 1.26 wt%, respectively (the loading amounts of MoS_2_ and RuO_2_ are calculated from the ICP results of Mo and Ru, respectively).

**Figure 5:**
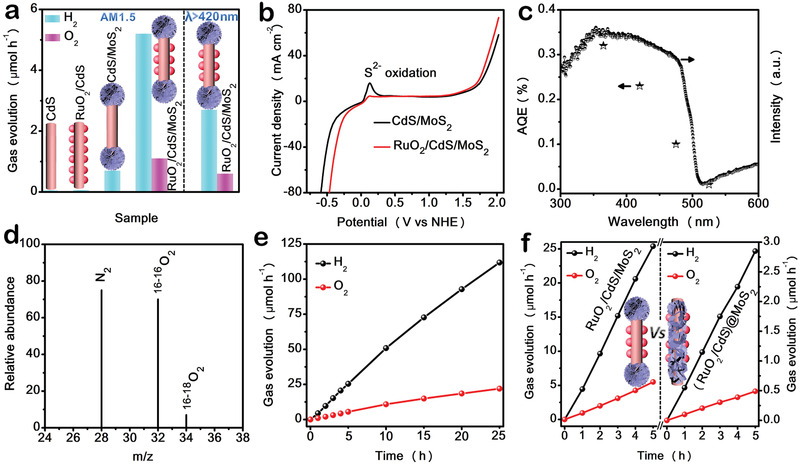
a) Gases evolution rate from water under the irradiation of a 300 W Xe lamp (AM1.5 air mass filter) catalyzed by CdS, RuO_2_/CdS, CdS/MoS_2_, and RuO_2_/CdS/MoS_2_, and gases evolution rate from water under the irradiation of visible light (*λ* >420 nm) catalyzed by RuO_2_/CdS/MoS_2_. b) LSV curves of CdS and RuO_2_/CdS/MoS_2_ in 1 m KOH electrolyte with a scanning rate of 50 mV s^−1^. c) AQE as a function of the irradiation wavelength in overall water‐splitting reaction over RuO_2_/CdS/MoS_2_. d) GC‐MS signals of the gas generated over RuO_2_/CdS/MoS_2_ for photocatalytic splitting of water containing 20% H_2_^18^O. e) The durable experiment of RuO_2_/CdS/MoS_2_. f) Time course of hydrogen and oxygen evolution under the irradiation of a 300 W Xe lamp (AM1.5 air mass filter) catalyzed by (RuO_2_/CdS)@MoS_2_ and RuO_2_/CdS/MoS_2_.

Although simultaneous H_2_ and O_2_ evolution based on RuO_2_/CdS/MoS_2_ nanodumbbells is achieved, the H_2_/O_2_ ratio deviated from the stoichiometric value of 2. To unravel the intrinsic reason, we used an iodometric method to detect potentially produced H_2_O_2_. As expected, with the water‐splitting proceeding, a trace amount of H_2_O_2_ was observed, and the concentration of H_2_O_2_ gradually increased over 5 h (Figure S16, Supporting Information), suggesting part of holes on the RuO_2_ sites take part in H_2_O_2_ evolution via 2‐eletrons pathway, which is supported by energy band structure (Figure S1d, Supporting Information). To further confirm that the detected O_2_ was indeed generated from H_2_O splitting, an isotopes labeling experiment was performed in a mixture of H_2_O and H_2_^18^O. As shown in Figure 5d, a signal at m/z = 34 is identified as ^16–18^O_2_, verifying that O_2_ is formed from photocatalytic overall water splitting.^[^ ^49^ ^]^ In addition, the stability experiment demonstrates negligible deterioration of photocatalytic activity for RuO_2_/CdS/MoS_2_ during 25 h irradiation (Figure 5e). SEM and TEM observations (Figure S17a–e, Supporting Information) show the recovered sample still retain the dumbbell morphology without obvious photocorrosion. The elemental composition and crystal structure remain unchanged, as confirmed by element mapping (Figure S17f, Supporting Information) and XRD pattern (Figure S18, Supporting Information), indicating the excellent stability of RuO_2_/CdS/MoS_2_. The apparent quantum efficiency (AQE) for RuO_2_/CdS/MoS_2_ during overall water splitting as a function of the incident light wavelength was displayed in Figure 5c. The AQE values for RuO_2_/CdS/MoS_2_ reach 0.32% at 365 nm, 0.23% at 425 nm, and 0.10% at 475 nm. When the light absorption at wavelengths is longer than 500 nm, the AQE value is nearly zero due to the low absorption efficiency for CdS in the visible‐light region beyond 500 nm.

To highlight the advantages of nanodumbbell structure with spatial separated sites in photocatalysis, we prepared (RuO_2_/CdS)@MoS_2_ core–shell photocatalyst with randomly loaded catalytic sites as control sample. As shown in Figure S19a (Supporting Information), RuO_2_ NPs are first deposited on the sidewalls of CdS nanowires and subsequently the obtained RuO_2_/CdS nanowires are wrapped by MoS_2_ NSs to form (RuO_2_/CdS)@MoS_2_ core–shell structure. SEM images (Figure S19b,c, Supporting Information), TEM images (Figure S19d–f, Supporting Information), and element mapping (Figure S19g,h, Supporting Information) indicate the successful preparation of (RuO_2_/CdS)@MoS_2_ core–shell structure. Besides, there are no distinct differences in light absorption (Figure S20a, Supporting Information), crystal structure (Figure S20b, Supporting Information), and the loading amount of dual cocatalysts (Table S2, Supporting Information) between (RuO_2_/CdS)@MoS_2_ and RuO_2_/CdS/MoS_2_, eliminating the effects of these three factors on photocatalytic performance. As indicated in Figure 5f, the rate of gas evolution for (RuO_2_/CdS)@MoS_2_ are almost 10 times faster than that of (RuO_2_/CdS)@MoS_2_ (0.57 µmol h^−1^ H_2_ and 0.1 µmol h^−1^ O_2_). The origin of the remarkably enhanced performance is attributed to the spatially separated catalytic sites for the H_2_ and O_2_ evolution. Basically, for RuO_2_/CdS/MoS_2_ dumbbell structure, the photogenerated electrons and holes have a remarkably tendency to locate at MoS_2_ and RuO_2_, respectively, thus facilitating charge separation. Nevertheless, regarding (RuO_2_/CdS)@MoS_2_, there are lots of contact interface between MoS_2_ and RuO_2_ due to their random loading (Figure S21, Supporting Information), which leads to the recombination of photoinduced charge carriers at the interfaces.

In conclusion, we demonstrated that a ternary RuO_2_/CdS/MoS_2_ photocatalyst with well‐defined dumbbell structure evolved H_2_ and O_2_ via a water‐splitting reaction under simulated solar light irradiation. The construction of spatially separated dual cocatalysts on CdS nanowires enables the photoexcited electrons and holes to transfer expeditiously from CdS to the MoS_2_ and RuO_2_ catalytic sites, respectively, resulting in a superior and durable photocatalytic water‐splitting activity. This work provides an efficient strategy for the design of metal‐sulfide‐based photocatalysts with high overall water‐splitting performance.

## Conflict of Interest

The authors declare no conflict of interest.

## Supporting information

Supporting Information
