# Supplementary material for: A Ternary Dumbbell Structure with Spatially Separated Catalytic Sites for Photocatalytic Overall Water Splitting
Source: Adv Sci (Weinh). 2020 Jul 14;7(17):1903568. doi: 10.1002/advs.201903568 (PMC7507026; doi:10.1002/advs.201903568)
Supplement: Supplementary file 1 — Supporting Information [file ADVS-7-1903568-s001.pdf]

Copyright WILEY-VCH Verlag GmbH & Co. KGaA, 69469 Weinheim, Germany, 2020.

Supporting Information

A Ternary Dumbbell Structure with Spatially Separated Catalytic Sites for Photocatalytic Overall Water Splitting

*Bocheng Qiu, Lejuan Cai, Ning Zhang, Xiaoming Tao and Yang Chai**

Experimental Section

Preparation of CdS nanowires: In a typical procedure, 125 mg of cadmium acetate and 75 mg thioacetamide were added into 40 mL ethylenediamine (EDA). After vigorously stirring for 1 h, the mixture was transferred into 50 mL Teflon-lined autoclave, sealed and heated at 200 °C for 18 h, and then allowed to cool to room temperature naturally. The finial product was obtained by centrifuging the suspension, washed with deionized (DI) water and alcohol in sequence at least three times, dried under vacuum at 45 ^{o}C overnight and finally treated in Ar at 400 ^{o}C for 2 h.

Preparation of CdS/MoS_2 nanodumbbells: In a typical procedure, the centrifugally collected CdS nanowires without washing treatment were dispersed in a mixed solvent containing 20 mL diethylenetriamine (DETA) and 4 mL DI water. After ultrasonic treatment for 15 min, 20 mg sodium molybdate and 80 mg thioacetamide were added into the solution. After stirring for 1 h, the solution was transferred into 50 mL Teflon-lined autoclave, sealed and heated at 200 °C for 18 h, and then allowed to cool to room temperature naturally. The resulting precipitates were collected by centrifuging, washed with ethanol and DI water three times until the organic molecules were completely removed, and then dried under vacuum at 45 ^{o}C overnight, and finally calcined in Ar at 400 ^{o}C for 2 h.

Preparation of RuO_2/CdS/MoS_2 nanodumbbells: In a typical procedure, the centrifugally obtained and unpurified CdS/MoS_2 nanodumbbells were dispersed in DI water under ultrasonic treatment. Then, a certain amount of ruthenium chloride was added into the above suspension. After stirring for 6 h, the dispersion was transferred into autoclave, sealed and heated at 150 °C for 6 h. After cooling to room temperature, the resulting precipitates were collected by centrifuged and washed, dried under vacuum, and finally calcined in Ar at 400 ^{o}C for 2 h to obtain RuO_2/CdS/MoS_2.

Preparation of RuO_2/CdS nanowires: In a typical procedure, the centrifugally collected CdS nanorods without washing treatment were dispersed in DI water under ultrasonic treatment. Then, a certain amount of ruthenium chloride was added into the above suspension. After stirring for 6 h, the dispersion was transferred into autoclave, sealed and heated at 150 °C for 6 h. After cooling to room temperature, the resulting precipitates were collected by centrifuged and washed, dried under vacuum, and finally calcined in Ar at 400 ^{o}C for 2 h to obtain RuO_2/CdS.

Preparation of RuO_2/CdS/MoS_2 with randomly loading cocatalysts: In a typical procedure, RuO_2/CdS were dispersed in 24 mL DI water. After ultrasonic treatment for 15 min, 20 mg sodium molybdate and 80 mg thioacetamide were added into the solution. After stirring for 1 h, the solution was transferred into 50 mL Tefon-lined autoclave, sealed and heated at 200 °C for 18 h, and then allowed to cool to room temperature naturally. The resulting precipitates were collected by centrifuging, washed with ethanol and DI water three times until the organic molecules were completely removed, dried under vacuum at 45 ^{o}C overnight, and calcined in Ar at 400 ^{o}C for 2 h.

Preparation of bare MoS_2: In a typical procedure, 20 mg sodium molybdate and 80 mg thioacetamide were added into a mixed solvent containing 20 mL diethylenetriamine (DETA) and 4 mL DI water. After stirring for 1 h, the solution was transferred into 50 mL Teflon-lined autoclave, sealed and heated at 200 °C for 18 h,

and then allowed to cool to room temperature naturally. The resulting precipitates were collected by centrifuging, washed with ethanol and DI water three times until the organic molecules were completely removed, and then dried under vacuum at 45 ^{o}C overnight, and finally calcined in Ar at 400 ^{o}C for 2 h.

Characterizations

X-ray diffraction (XRD) patterns of all samples were collected in the range 10-80° (2θ) using a RigakuD/MAX 2550 diffract meter (Cu K radiation, λ =1.5406 Å), operated at 40 kV and 100 mA. The morphologies were characterized by transmission electron microscopy (TEM, JEM2011EX and JEM 2100F). The composition of all the samples was characterized by EDX attached to the HRTEM. HAADF-STEM image and elemental mapping were collected using a TEM (JEOL, JEM-2100F) equipped with EDX spectroscopy. ICP analysis was conducted on a Vista Axial (USA) spectrometer. The surface morphologies were observed by field emission scanning electron microscopy (TESCAN MAIA3). The instrument employed for XPS studies was a Perkin-Elmer PHI 5000C ESCA system with Al Kα radiation operated at 250 W. The shift of the binding energy due to relative surface charging was corrected using the C1s level at 284.4 eV as an internal standard. Fourier transform infrared (FTIR) spectra were recorded with attenuated total reflection (ATR) mode containing the powder sample with the FTIR spectrometer (VERTEX 70v, Bruker). The photoluminescence (PL) spectra were carried out by a Hitachi F-4600 fluorescence spectrophotometer at room temperature and excited by an incident light of 360 nm. Photoelectrochemistry measurements were performed on a standard three electrode cell with a working electrode, a graphite carbon as the counter electrode, and a saturated calomel electrode as the reference electrode under Xe light irradiation. The working electrode was prepared through a clean fluoride-tin oxide deposited with a sample film. The aqueous solution of 0.5 m Na_2SO_4 purged with nitrogen gas was used as the electrolyte. The EIS measurements were performed in a 25 mM $K_3Fe(CN)_6$ and $K_4Fe(CN)_3$ and 0.1 M KCl mixture aqueous solution.

Photocatalytic activity test.

The photocatalytic water splitting experiments were conducted on an outer irradiation-type photoreactor at ambient temperature (5 °C) using a 300 W Xe lamp equipped with an AM 1.5 solar simulator or a UV-cutoff filter ($\lambda \geq 420$ nm). 100 mg of catalyst was dispersed 100 mL of water, and then the suspension was stirred and vacuumized for 30 min. Gas evolution was observed only under irradiation, being analyzed by an online gas chromatograph (GC, TCD, nitrogen as a carrier gas and 5 Å molecular sieve column). The durable water splitting experiment is up to 25 h. The isotope labelling experiments were conducted in a sealed Pyrex flask. Typically, the powder sample was dispersed into aqueous environment containing 20% $H_2^{18}O$ with ultrasonic treatment. After Xe lamp irradiation for 5 h, the gas mixture was analyzed using Gas Chromatography-Mass Spectrometer (GC-MS) (Agilent Technologies 5975C Mass Selective Detector) operated in selective-ion mode to monitor for N_2, $^{16\text{-}16}O_2$, $^{16\text{-}18}O_2$, and $^{18\text{-}18}O_2$ ions.

Apparent quantum yield (AQY) measurements.

The AQY of the photocatalytic overall water splitting was performed by using a 300 W Xe lamp equipped with $\lambda \pm 20$ nm band-pass filter and calculated by the following formula:

$$AQY = \frac{\text{N} * \text{the number of the evolved gas molecules}}{\text{The number of incident photons}}$$

N represents the number of electrons used to generate one molecule of H_2 or O_2, that is, 2 or 4 for one molecule of H_2 or O_2 evolution, respectively.

Selective photodeposition of Au or MnO_x

For Au selective deposition, 50 mg $RuO_2/CdS/MoS_2$ was dispersed into 100 mL 5% lactic acid, and then a certain amount of $HAuCl_4$ solution was added into the above mixture under stirring. The suspension was irradiated under a 300 W Xe lamp equipped with a UV-cutoff filter ($\lambda \geq 420$ nm). After 30 min photoreduction, the sample was collected by centrifuging, washed with ethanol and DI water three times.

For MnO_x selective deposition, 50 mg RuO_2/CdS/MoS_2 was dispersed into 100 mL 20mM $NaIO_3$ solution, and then a certain amount of $MnCl_2$ solution was added into the above mixture under stirring. The suspension was irradiated under a 300 W Xe lamp equipped with a UV-cutoff filter (λ≥420 nm). After 30 min photoxidation reaction, the sample was collected by centrifuging, washed with ethanol and DI water three times.

Detection of H_2O_2 over RuO_2/CdS/MoS_2

The content of H_2O_2 was measured by an iodometric method. In typical, 5 mL supernatant was added into the mixture of 100 mM potassium iodide and 10 mM ammonium molybdate tetrahydrate. Then the mixed solution stands for a long time, and the absorption peak of iodine in 352 nm was applied to detect its concentration by using a UV-vis spectrophotometry.

Computational Methods

The spin-polarized first-principles calculations based on the Density Functional Theory (DFT) were performed using the Vienna *Ab initio* Simulation Package (VASP).[1] The Perdew-Burke-Ernzerhof (PBE) exchange-correlation functional[2] within the generalized gradient approximation (GGA) was employed to describe the exchange-correlation energy. The projector-augmented-wave (PAW)[3] method was adopted for the pseudopotentials. The energy cutoff for the plane wave basis expansion was set to 450 eV. One monolayer of MoS_2 (2H) and a layer of RuO_2 (110) slab were respectively put on the $(10\bar{1}1)$ plane of CdS with the thickness of about 15.45 Å to construct the hetreostructures. A vacuum layer of 15 Å in the z direction to avoid the interaction between layers. The lateral lattice constants and the atom positions were fully optimized with the force on each atom of 0.02 eV/Å for convergence criterion. The sampling in the Brillouin zone was set with 3×2×1 by the Monkhorst-Pack method.[4]

Femtosecond transient absorption (fs-TA) measurements. The ultrafast TA experiments were performed at room temperature on using a pump-probe laser system (FemPum) based on a regenerative amplified Ti: sapphire laser system from Coherent (800 nm, 35 fs, and 1 kHz repetition rate). The pump pulses at 400 nm were produced by a BBO crystal as a second harmonic of the laser. The white-light continuum probe pulses (450-700 nm) were generated by focusing 800 nm pulses (0.1 mJ) into a 2.0 mm thick sapphire window. The data were finally analyzed through the commercial software (Surface Xplorer, Ultrafast Systems).

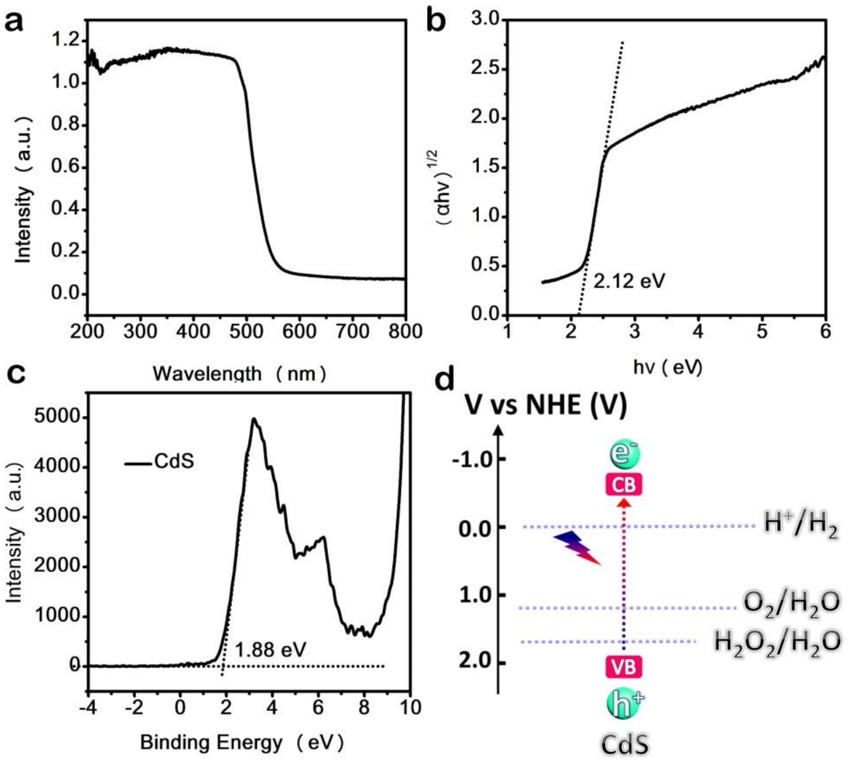

Figure S1. (a) UV-vis absorption spectrum, (b) $(\alpha h\nu)^{1/2}$ versus hν curve, (c) VB-XPS spectrum of CdS nanowires, and (d) band structure diagram of CdS nanowires. VB, valence band; CB, conduction band.

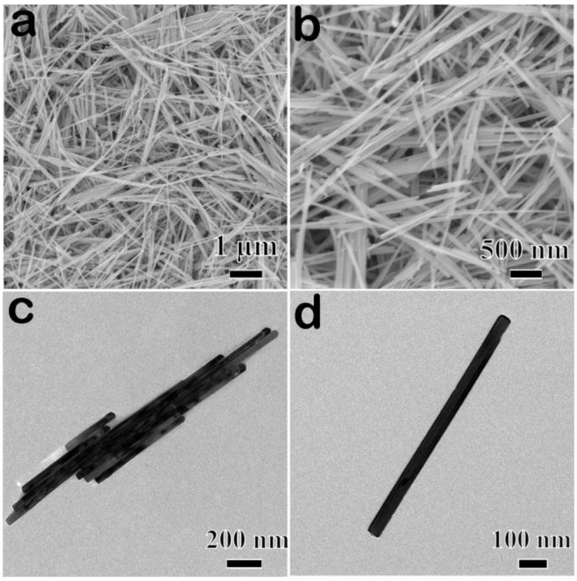

Figure S2. (a,b) SEM and (c,d) TEM images of CdS nanowires.

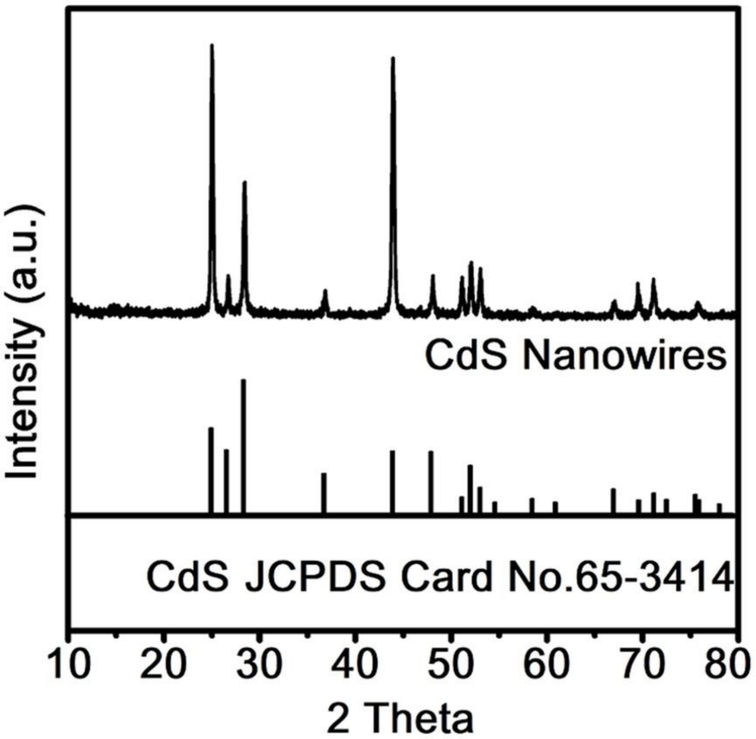

Figure S3. XRD pattern of CdS nanowires.

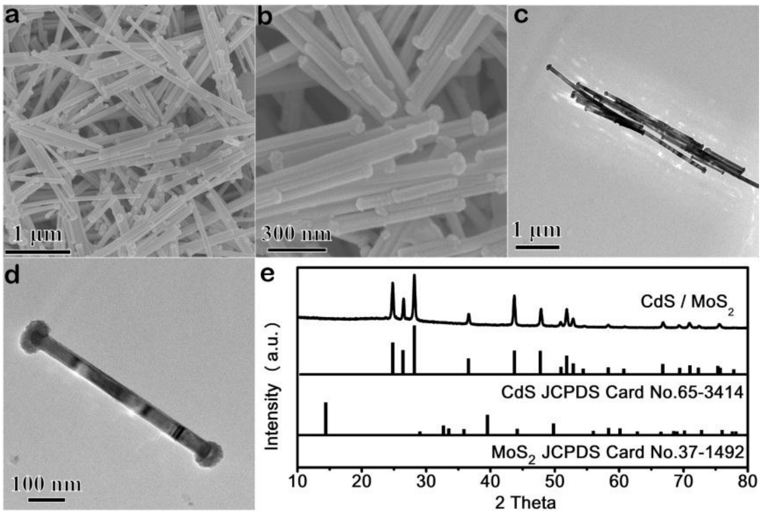

Figure S4. (a,b) SEM images, (c,d) TEM images, and (e) XRD pattern of CdS/MoS_2 nanodumbbells.

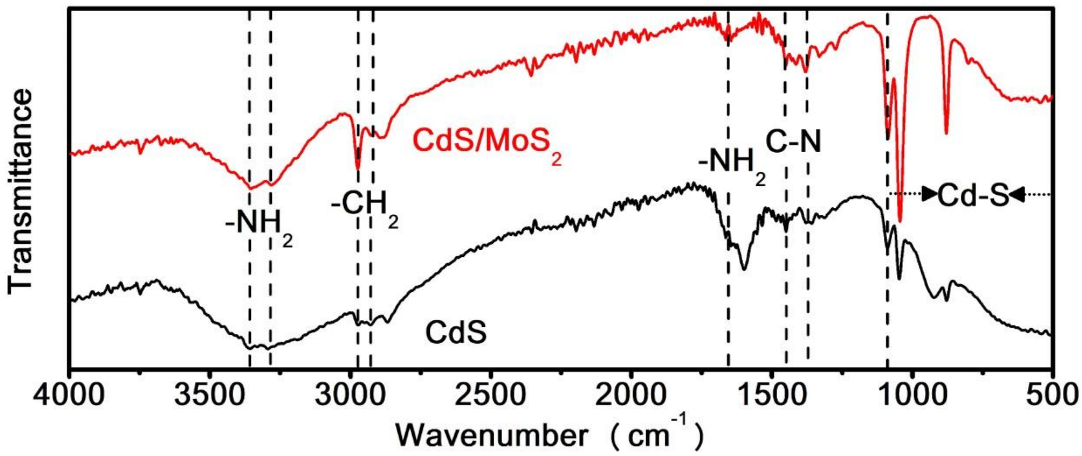

Figure S5. FT-IR spectra of CdS nanowires and CdS/MoS_2 nanodumbbells.

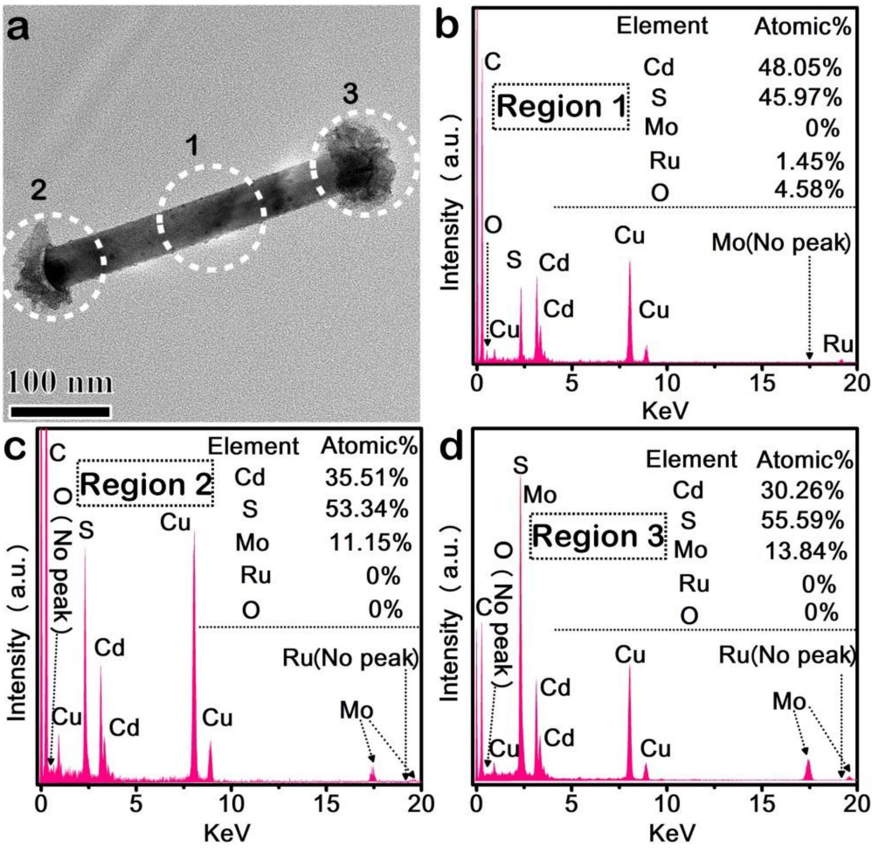

Figure S6. (a) TEM image of single $RuO_2/CdS/MoS_2$ nanodumbbell. (b,c,d) EDX spectra for the selected regions (1,2,3).

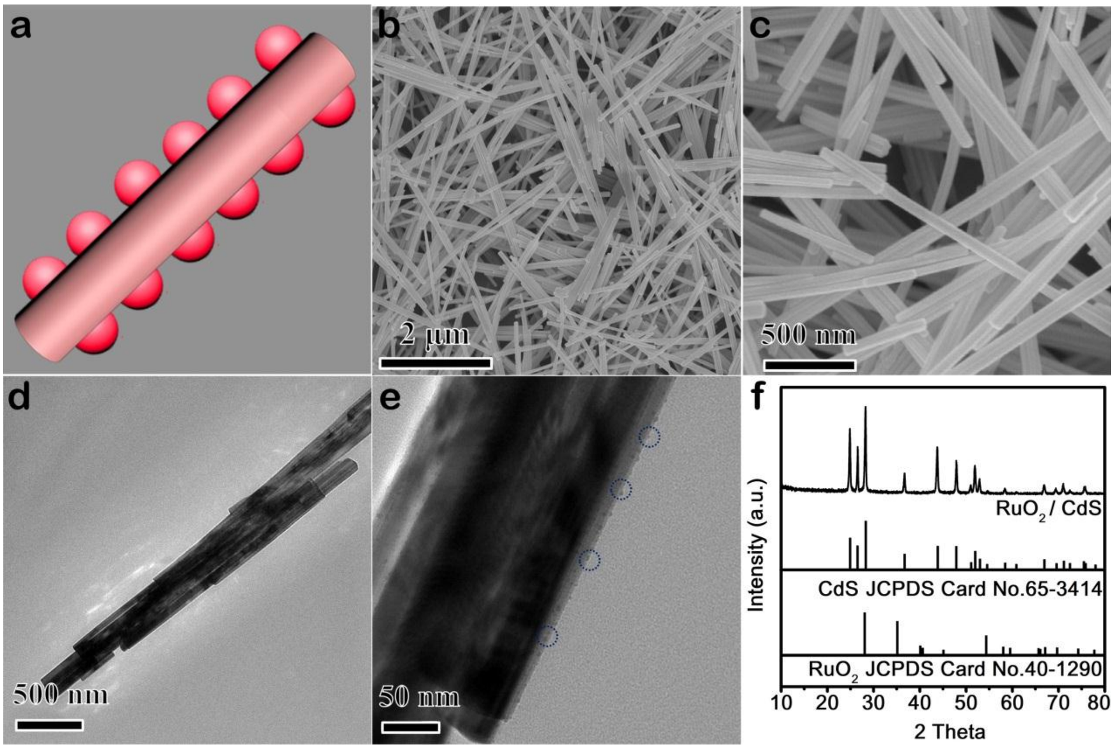

Figure S7. (a) RuO_2/CdS NWs morphologic diagram. (b,c) SEM images, (d,e) TEM images, and (f) XRD pattern of RuO_2/CdS nanowires.

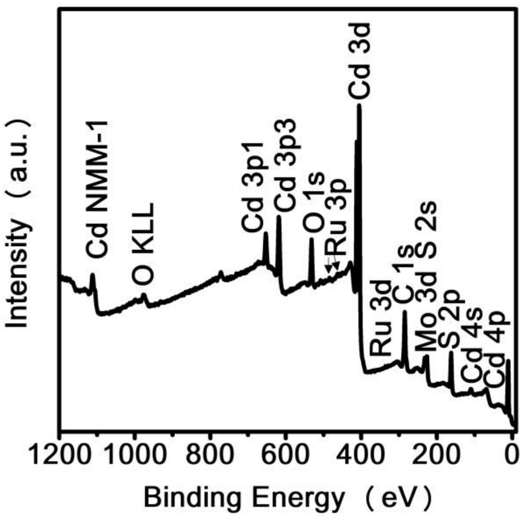

Figure S8. XPS survey spectrum of $RuO_2/CdS/MoS_2$ nanodumbbells.

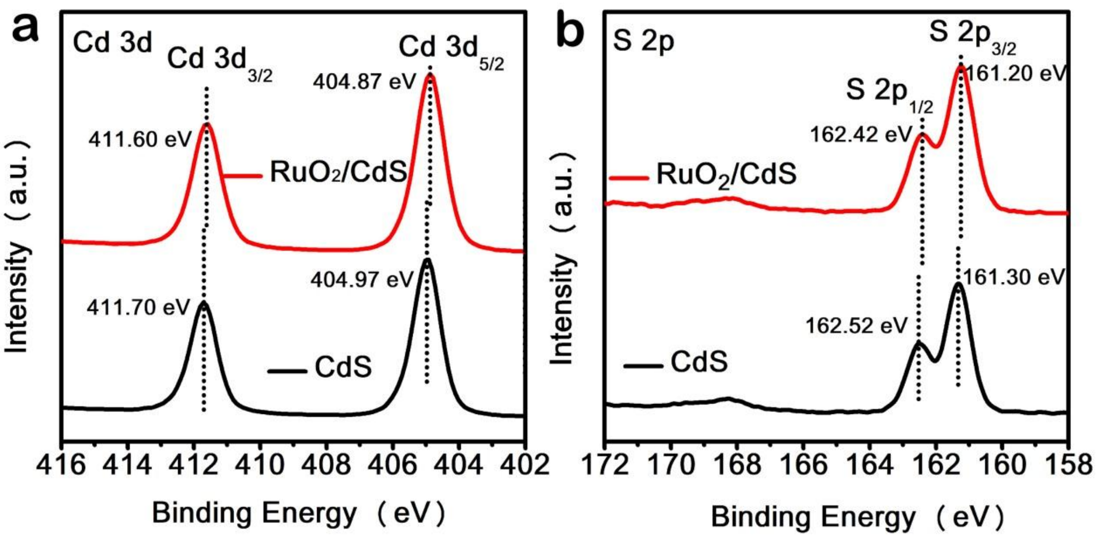

Figure S9. High resolution XPS spectra of Cd 3d (a) and S 2p (b) of CdS and RuO_2/CdS.

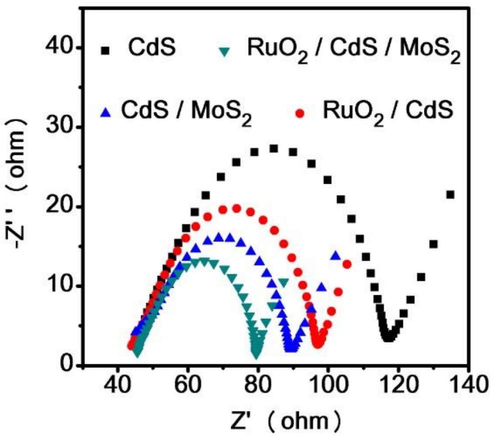

Figure S10. EIS spectra of CdS, RuO_2/CdS, CdS/MoS_2, and RuO_2/CdS/MoS_2.

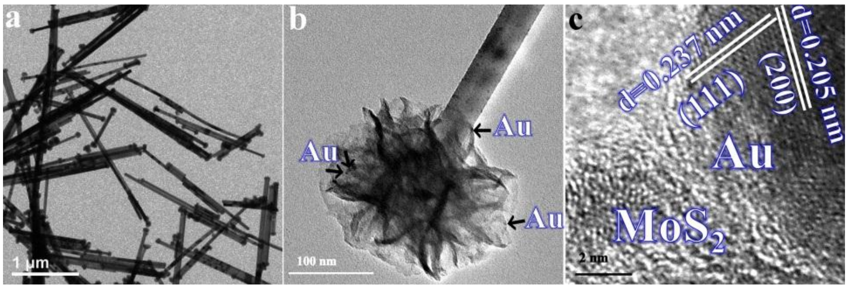

Figure S11. (a,b) TEM and (c) HRTEM images of $RuO_2/CdS/MoS_2$ after the photodeposition of Au nanoparticles.

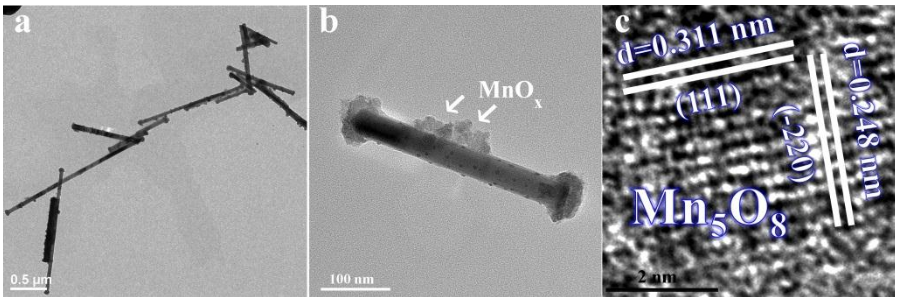

Figure S12. (a,b) TEM and (c) HRTEM images of RuO_2/CdS/MoS_2 after the photodeposition of MnO_x nanosheets.

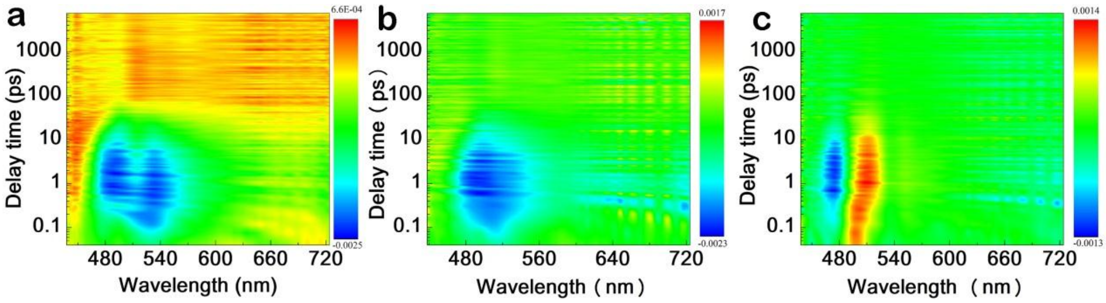

Figure S13. 2D pseudo-color plot of TA spectra of (a) CdS, (b) CdS/MoS_2, and (c) $RuO_2/CdS/MoS_2$.

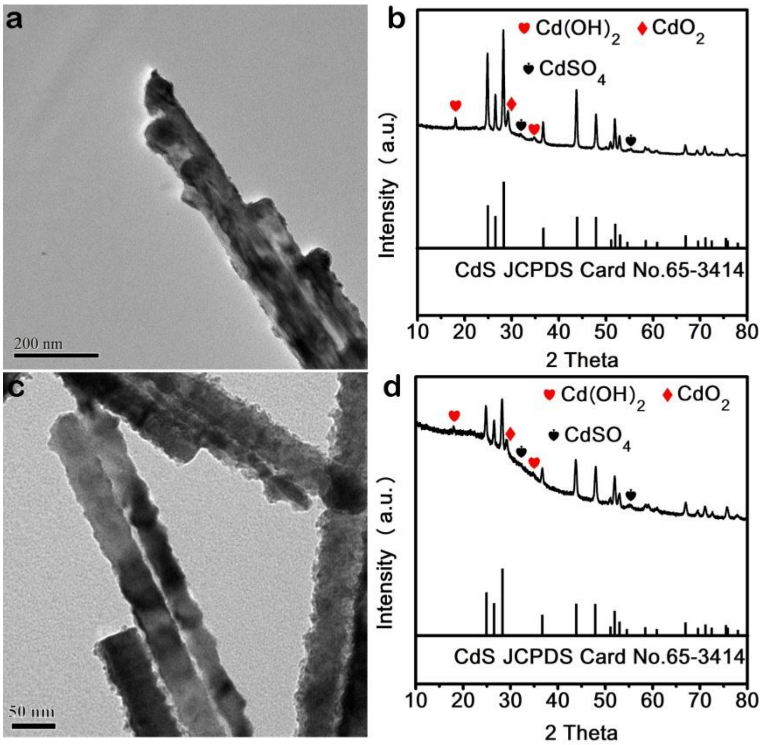

Figure S14. (a) TEM image and (b) XRD patterns of CdS/MoS_2; (c) TEM image and (d) XRD patterns of CdS;

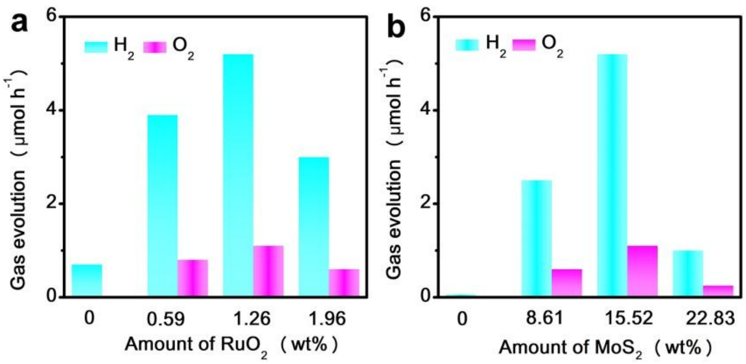

Figure S15. The effect of amounts of (a) RuO_2 and (b) MoS_2 on photocatalytic activity of RuO_2/CdS/MoS_2 for overall water splitting. The loading amounts are 15.52 wt% for MoS_2 in (a), and 1.26 wt% for RuO_2 in (b)

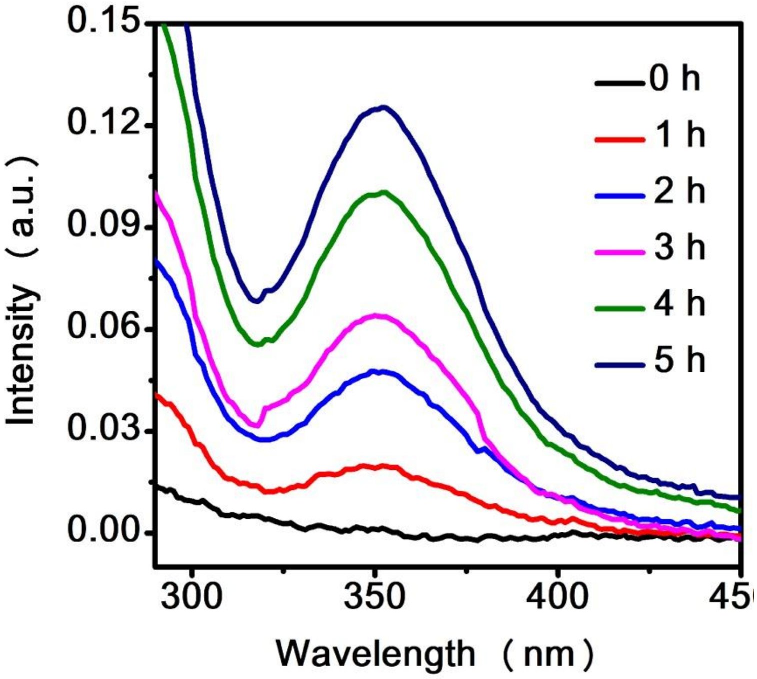

Figure S16. Time dependence of the absorption intensity of H_2O_2 over RuO_2/CdS/MoS_2 during photocatalytic water splitting; the content of H_2O_2 was measured by an iodometric method.

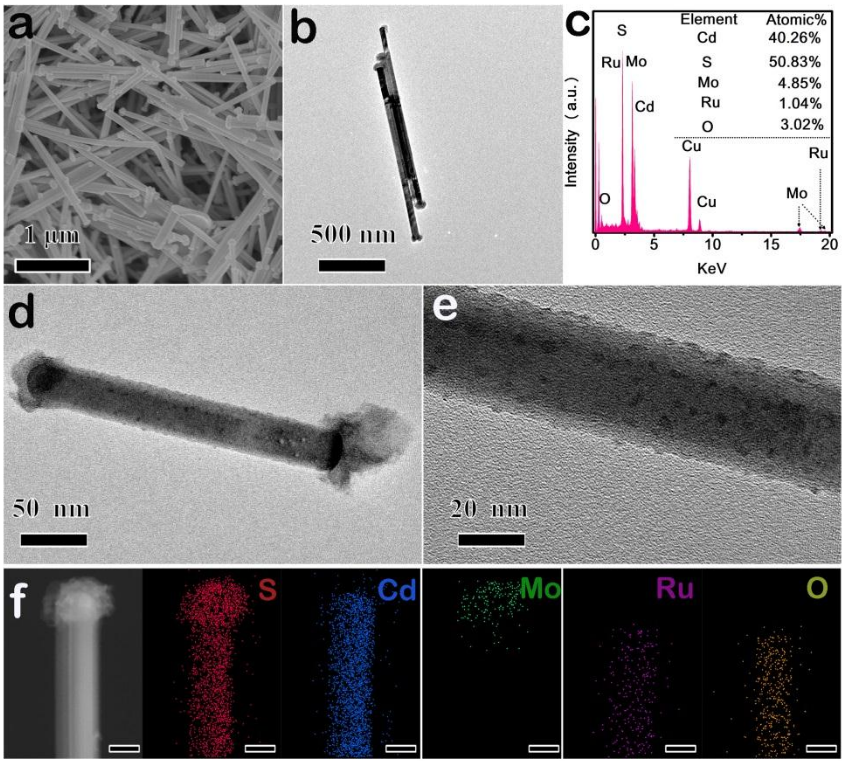

Figure S17. (a) SEM images, (b) TEM images, and (c) EDX spectrum of $RuO_2/CdS/MoS_2$ nanodumbbells. (d,e) HRTEM images of $RuO_2/CdS/MoS_2$ nanodumbbells. (f) Element mapping of S, Cd, Mo, Ru, O. The scar bar is 50 nm.

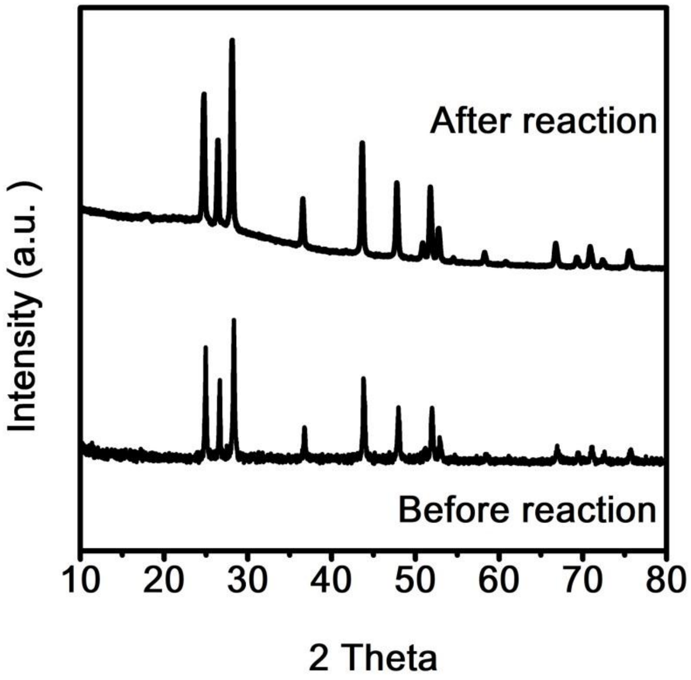

Figure S18. XRD patterns of $RuO_2/CdS/MoS_2$ before and after water splitting.

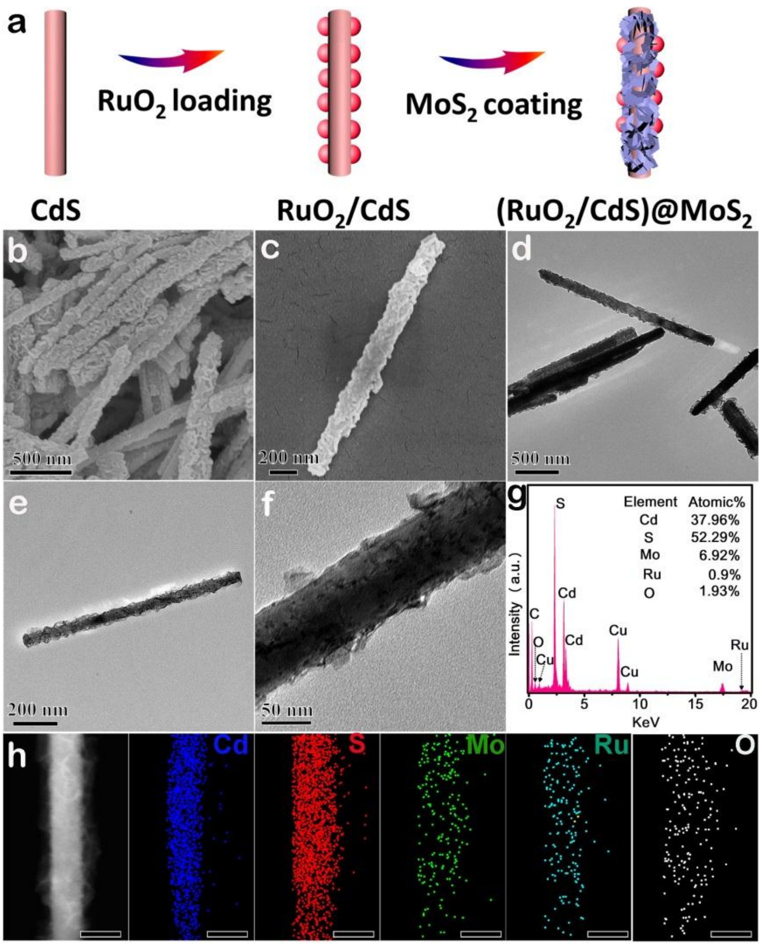

Figure S19. (a) Schematic illustration of $(RuO_2/CdS)@MoS_2$ preparation process. (b,c) SEM images and (d,e,f) TEM images of $(RuO_2/CdS)@MoS_2$. (g) EDX spectrum and (h) the corresponding element mapping of S, Cd, Mo, Ru, O. The scar bar is 50 nm.

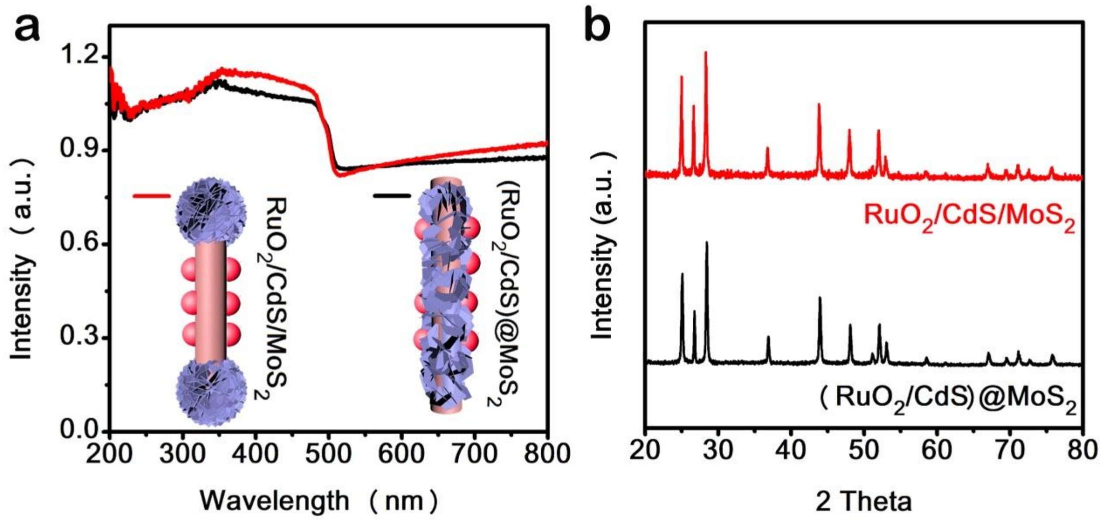

Figure S20. (a) UV-vis absorption spectra and (b) XRD patterns of $(RuO_2/CdS)@MoS_2$ and $RuO_2/CdS/MoS_2$

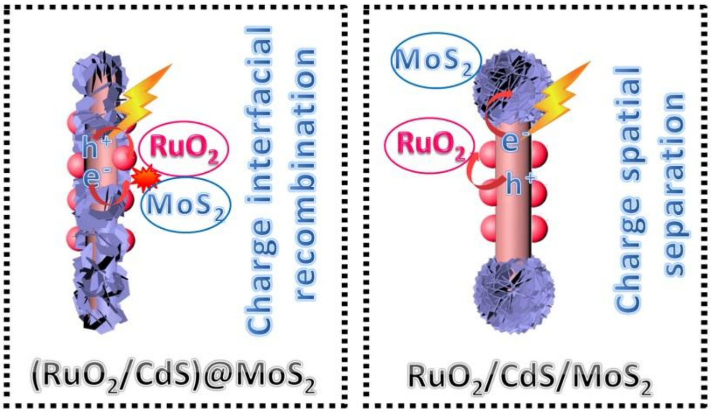

Figure S21. Schematic representation of proposed charge transfer **routes in** $(RuO_2/CdS)@MoS_2$ and $RuO_2/CdS/MoS_2$.

Table S1. Summary of ICP results of RuO_2/CdS/MoS_2 and (RuO_2/CdS)@MoS_2.

Sample	Measured content of Mo (wt%)	Measured content of Ru (wt%)
RuO_2/CdS/MoS_2	9.31	0.96
(RuO_2/CdS)@MoS_2	8.94	1.05

Table S2. Bi-exponential fits for the fs-TA decay curves of CdS, CdS/MoS_2, and RuO_2/CdS/MoS_2 under 400 nm excitation.

Sample	A_1	τ_1 (ps)	A_2	τ_2 (ps)
CdS	0.92	7.8	0.08	350.8
CdS/MoS_2	0.88	8.1	0.12	123.5
RuO_2/CdS/MoS_2	0.83	8.3	0.17	40.8

Reference

[1] G. Kresse, J. Furthmuller, Comput. Mater. Sci. 1996, 6, 15; G. Kresse, J. Furthmuller, Phy. Rev. B 1996, 54, 11169; G. Kresse, J. Hafner, Phys. Rev. B 1994, 49, 14251.

[2] J. Perdew, J. A. Chevary, S. H. Vosko, K. Jackson, M. Pederson, D. J. Singh, C. Fiolhais, Phys. Rev. B 1992, 46, 6671.

[3] P. E. Blochl, Phys. Rev. B 1994, 50, 17953.

[4] H. J. Monkhorst, J. D. Pack, Phys. Rev. B 1976, 13, 5188.
